# Supplementary material for: Characterization of a Droplet Containing the Clustered Magnetic Beads Manipulation by Magnetically Actuated Chips
Source: Micromachines (Basel). 2022 Sep 28;13(10):1622. doi: 10.3390/mi13101622 (PMC9610654; doi:10.3390/mi13101622)
Supplement: Supplementary file 1 [file micromachines-13-01622-s001.zip › supplementary files/Supplementary Document S1.pdf]

Supplementary file S1

Consider a droplet that wetted on a surface due to surface tension. For simplicity, the wetting process assumes that the droplet remains spherical (Fig. S1). In the case of a spherical droplet, the relation between volume and diameter (d_D) is as follows:

$$V_D = \frac{\pi}{6} d_D^3 \tag{S1}$$

Here V_D is the volume of the droplet.

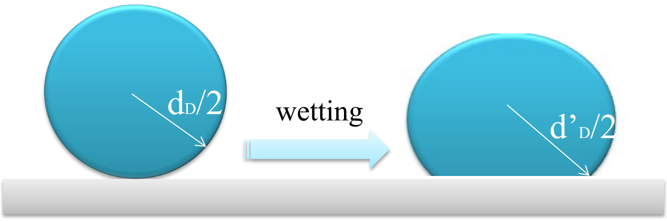

Figure S1. An illustration of a spherical droplet is wetting on the substrate.

For a wetting droplet illustrated in Fig. S2, the volume (V_D), side surface area ($A_{D,s}$), and base surface area ($A_{D,b}$) with the spherical shape can be expressed as follows:

$$V_D' = \int_{\pi-\theta}^{\pi} \int_0^{2\pi} \int_0^{R_D'} r^2 \sin\phi dr d\varphi d\phi = \frac{2\pi R_D'^3 (1-\cos\theta)}{3} = \frac{\pi d_D'^3 (1-\cos\theta)}{12} \tag{S2}$$

$$A_{D,S}' = \int_{\pi-\theta}^{\pi} \int_0^{2\pi} R_D'^2 \sin\theta d\varphi d\phi = 2\pi R_D'^2 (1-\cos\theta) = \frac{\pi d_D'^2 (1-\cos\theta)}{4} \tag{S3}$$

$$A_{D,b}' = \int_0^{R_D' \sin\theta} \int_0^{2\pi} r dr d\varphi = \pi R_D'^2 \sin^2\theta = \frac{\pi d_D'^2 \sin^2\theta}{4} \tag{S4}$$

where d'_D is wetting diameter of a droplet.

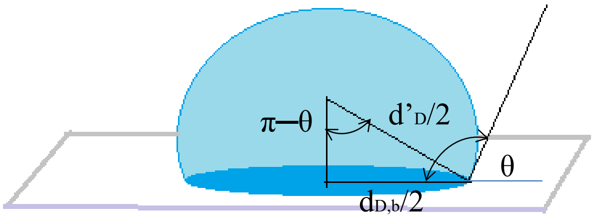

Figure S2. Schematic diagram of a droplet characterized by diameters of d'_D, $d_{D,b}$ and contact angle θ.

By substituting Eq.(S-1) into Eq.(S-2), the diameter (d'_D) of a wetting droplet is obtained as:

$$d_D' = \left(\frac{2}{(1-\cos\theta)}\right)^{1/3} d_D \tag{S5}$$

According to the following expression, the droplet's base diameter ($d_{D,b}$) is wetted with respect to the diameter (d'_D) as shown in Fig. S3:

$$d_{D,b} = d'_D \sin(\pi-\theta) = \left(\frac{2\sin^3\theta}{(1-\cos\theta)}\right)^{1/3} d_D \tag{S6}$$

The frictional force (Fμ) between a liquid droplet and a substrate surface can be expressed as the difference between gravitational force and buoyance force:

$$F_{\mu} = \mu_k(\rho_W V_D g - \rho_O V_D g) = \mu_k \Delta\rho V_D g \tag{S7}$$

where μ_k is friction coefficient of the substrate surface. $\Delta\rho$ is the difference density between liquid droplets and surrounding medium. g is gravitational acceleration. Upon moving onto a substrate, a droplet's surface tension is expressed in the following manner:

$$F_{\gamma} = \pi d_{D,b} \gamma_{O-W} \cos\theta \tag{S8}$$

where θ is the contact angle. $\gamma_{O\text{-}W}$ is the relative surface tension between liquid water and oil medium. When the droplet moves, determining the frictional force can be a challenge. Therefore, surface tension is used instead. In order to present the ratio between frictional force and surface tension force, the coefficient of ζ is introduced to define as follows::

$$\zeta = \frac{F_f}{F_{\gamma}} = \frac{\mu_k \Delta\rho \frac{\pi}{6} d_D^3 g}{\pi d_{D,b} \gamma_{O-W} \cos\theta} \tag{S9}$$

By substituting Eq.(S-5) into Eq.(S-9) , the equation can be obtained as follows:

$$\zeta = \frac{\mu_k \Delta\rho \frac{\pi}{6} d_D^3 g}{\pi \left(\frac{2\sin^3\theta}{(1-\cos\theta)} \right)^{1/3} d_D \gamma_{O-W} \cos\theta} \tag{S10}$$

$$= \frac{\mu_k \Delta\rho \left(\frac{6V_D}{\pi} \right)^{2/3} g}{6\gamma_{O-W} \left(2\sin^3\theta \cos^3\theta / (1-\cos\theta) \right)^{1/3}} \tag{S11}$$

$$= \left(\frac{4(1-\cos\theta)}{6\pi^2 \sin^3 2\theta} \right)^{1/3} \frac{\mu_k \Delta\rho (V_D)^{2/3} g}{\gamma_{O-W}} \tag{S12}$$

$$= \left(\frac{2(1-\cos\theta)}{3\pi^2 \sin^3 2\theta} \right)^{1/3} \frac{\mu_k \Delta\rho (V_D)^{2/3} g}{\gamma_{O-W}}$$

In this study, the value of ζ is calculated to be 0.001 while μ=0.05, $\Delta\rho$=60 kg/m^3, V_D=20 μL, $\gamma_{O\text{-}W}$=0.051 N/m, g=9.81 m/s^2, θ=110°.
